# Evidence-based practice in traditional persian medicine (TPM): a stakeholder and social network analysis

**DOI:** 10.1186/s12906-024-04564-5

**Published:** 2024-07-03

**Authors:** Seyed Reza Abdipour Mehrian, Shahadat Uddin, Zahra Ghahramani, Reza Moshfeghinia, Saeed Shahabi, Aliakbar Haghdoost, Golsa Mesbahi, Mahmoud Khodadost, Mohammad Hashem Hashempur, Mojtaba Heydari, Morteza Mojahedi, Majid Nimrouzi, Mehdi Pasalar, Hossein Molavi Vardanjani, Kamran Bagheri Lankarani

**Affiliations:** 1 Health Policy Research Center, Institute of Health, Shiraz University of Medical Sciences, Shiraz, Iran; 2 Complex Systems Research Group, Faculty of Engineering & IT, The University of Sydney, Darlington, Australia; 3 Hematology Research Center, Shiraz University of Medical Sciences, Shiraz, Iran; 4 Student Research Committee, Shiraz University of Medical Sciences, Shiraz, Iran; 5 Institute for Futures Studies in Health, Kerman University of Medical Sciences, Kerman, Iran; 6 Beheshti Shahid University of Medical Sciences, Medicine Traditional Iranian of Department, Tehran, Iran; 7 Research Center for Traditional Medicine and History of Medicine, Department of Persian Medicine, School of Medicine, Shiraz University of Medical Sciences, Shiraz, Iran; 8 Research Center for Traditional Medicine and History of Medicine, Shiraz University of Medical Sciences, Shiraz, Iran; 9 Traditional Medicine and History of Medical Sciences Research Center, Babol University of Medical Sciences, Babol, Iran; 10 Essence of Parsiyan Wisdom Institute, Research Center for Traditional Medicine and History of Medicine, Shiraz University of Medical Sciences, Shiraz, Iran; 11 MD-MPH Program, School of Medicine, Research Center for Traditional Medicine and History of Medicine, Shiraz University of Medical Sciences, Shiraz, Iran

**Keywords:** Stakeholder, Evidence-based practice, Traditional persian medicine, Social network analysis

## Abstract

**Background:**

The utilization of complementary and alternative medicine (CAM) is experiencing a global surge, accompanied by the adoption of national CAM policies in numerous countries. Traditional Persian medicine (TPM) is highly used as CAM in Iran, and the ongoing scientific evaluation of its interventions and the implementation of evidence-based medicine (EBM) encounters various barriers. Therefore, comprehending the characteristics and interactions of stakeholders is pivotal in advancing EBM within TPM policies. In this study, we utilized both classical stakeholder analysis and social network analysis to identify key stakeholders and potential communication patterns, thereby promoting EBM in TPM policy-making.

**Methods:**

A cross-sectional nationwide stakeholder analysis was conducted in 2023 using snowball sampling. The interviews were carried out using a customized version of the six building blocks of health. Data were collected through semi-structured interviews. Stakeholders were assessed based on five factors (power, interest, influence, position, and competency). The connections and structure of the network were analyzed using degree, betweenness, closeness centrality, and modularity index to detect clusters of smaller networks.

**Results:**

Among twenty-three identified stakeholders, the Ministry of Health and Medical Education (MOHME) and the Public were the most powerful and influential. The Iranian Academy of Medical Sciences was the most competent stakeholder. Social network analysis revealed a low density of connections among stakeholders. Pharmaceutical companies were identified as key connectors in the network, while the Public, supreme governmental bodies, and guilds acted as gatekeepers or brokers. The MOHME and Maraji were found to be high-ranking stakeholders based on four different centrality measures.

**Conclusion:**

This study identifies powerful stakeholders in the network and emphasizes the need to engage uninterested yet significant stakeholders. Recommendations include improving competence through education, strengthening international relations, and fostering stronger relationships. Engaging key connectors and gatekeepers is essential for bridging gaps in the network.

**Supplementary Information:**

The online version contains supplementary material available at 10.1186/s12906-024-04564-5.

## Introduction

The utilization of Traditional and Complementary Medicine (T&CM), a subset of Complementary and Alternative Medicine (CAM), has seen a remarkable global surge. In 2018, 88% of WHO member countries reported its use. Over the years, there has been a significant increase in the number of countries with national T&CM policies, rising from 25 in 1999 to 98 in 2018. Moreover, since 2005, there has been a significant improvement in the regulation and registration of herbal medicines in the WHO Eastern Mediterranean Region. Nine of its 21 nations now have national T&CM policies, and 12 have laws and regulations related to T&CM [1].

In the Middle Eastern region, Iran boasts a rich history of traditional medicine, with Traditional Persian medicine (TPM) standing out as a prominent example. Given the increasing global trend of CAM adoption, it is crucial to examine the current state of TPM in Iran, especially in light of the country’s efforts to integrate evidence-based medicine (EBM) into its healthcare system. Although TPM has a long-established history in Iran, the scientific evaluation of its interventions is a recent development, requiring further progress.

Evidence-based medicine (EBM) involves using the current best evidence to make decisions about individual patient care [2]. Although EBM has been considered for implementation in patient care in many medical science areas in Iran [3], barriers to its implementation exist. These barriers encompass inadequate facilities [4], research-related issues, and problems with coordination and motivation [5].

Integrating EBM into every aspect of the health system is crucial, but its integration into CAM practice is particularly significant due to the high demand and the relatively low level of scientific evaluation of CAM interventions. Recognizing this need, changes have been implemented in the healthcare system. In 2007, TPM was officially integrated into the Iranian healthcare system, with major medical universities establishing Schools of Persian Medicine and allied divisions. This allowed doctors of medicine and pharmacy to specialize in Persian medicine and traditional pharmacy at the Ph.D. level [6].

The widespread use of TPM in Iran, alongside conventional medicine [7–10]. has led to the participation of various groups in delivering traditional treatments. These include unlicensed therapists and natural remedy shops, drawn by financial advantages. Legally-approved service providers encompass TPM clinics, specialized private offices, and traditional and herbal drug manufacturers [6].Despite the existing controversies, CAM interventions are frequently utilized in Iran, with up to 75% of outpatients [11] and over 50% of cancer patients relying on at least one CAM method [12]. Medicinal plants, TPM, hydrotherapy, and music therapy are common treatment methods [7, 13]. However, studies indicate that fewer than 12% of CAM users receive guidance from approved therapists, raising concerns about the potential misuse of CAM [6, 7]. Non-EBM approaches to CAM can also be detrimental to health [14], and the unclear identification, contamination, and adulteration of medicinal plants are among the disadvantages of such approaches [15]. Examples of harm resulting from non-EBM evidence-based medicine include hepatic failure and hospital admissions due to borage (Echium amoenum) misuse [16].

Over the past two decades, the number of published articles on EBM in Iran has increased. However, few studies have examined the existing policies and policy processes. Therefore, the need to improve the approach to EBM in medical sciences remains necessary, particularly with the growing use of TPM. Considering that decision-making processes are influenced by stakeholder characteristics, understanding these stakeholders, their features, and how they interact with and influence each other is crucial for facilitating the use of EBM [17].

This study aimed to identify the key stakeholders in TPM, their characteristics, and their relationships in Iran, where TPM was the most common CAM practice. Using social network analysis (SNA), we visualized and analyzed stakeholder connections and communication patterns to identify key stakeholders and their roles in the policy process, as well as potential communication gaps.

## Methods

### Design

Our cross-sectional study was conducted in Iran between 2022 and 2023, and it involved a stakeholder analysis conducted in three phases. Firstly, a list of potential stakeholders was generated by reviewing available literature and information on the Internet. Secondly, semi-structured interviews of 45 min to 1 h were conducted with 24 individuals (see Supplementary Table 1), using a framework modified from the six building blocks framework [18] to categorize different aspects of TPM practice at various levels (see Supplementary Table 2). Finally, interviewees were asked to assess the stakeholders based on five factors (power, interest, influence, position, and competency), and the stakeholders with the highest scores were included in the network analysis. A panel of experts who were previously interviewed defined the connections between key stakeholders.

### Sampling and data collection

We recruited informed stakeholders of TPM using convenience and snowball sampling methods. Interviews continued until data saturation was achieved. The interviews began with a description of the study objectives, after which experts were asked, “Who are the main actors involved in the decision-making and policy-making processes of organizing evidence-based traditional Persian medicine in Iran?“. Using the modified tool, the interviewees also were questioned about various aspects of TPM practice at different levels (see Supplementary Table 2). Subsequently, the interviews were transcribed, and a list of individuals and organizations mentioned was generated. Similar or subordinate individuals and organizations were consolidated by an expert panel. Different subordinates of the Ministry of Health and Medical Education (MOHME) were analyzed separately due to their critical roles in Iran’s health system. The final stakeholders were evaluated by an expert panel consisting of specialists in TPM, conventional medicine physicians, and health policymakers. Stakeholders were assessed based on power, interest, influence, position, and competency. Each stakeholder received a score from 0 to 10, with 0 indicating the lowest level of the feature and 10 indicating the highest. A score of 0 represented complete opposition to the policy, 5 indicated neutrality, and 10 signified complete support for the policy. Position scores were recalculated to account for highly opposing stakeholders (|Position-5|×2), and those with the highest scores were included in the SNA. The expert panel subsequently rated the connections of the final stakeholders using a four-level scale: no connection, weak connection, moderate connection, and strong connection.

### Definitions and rationale

The five factors were selected after an extensive literature review on stakeholder analysis, as they are vital for identifying and comprehending the roles and behavior of stakeholders in healthcare policy-making and implementation [17, 19, 20]. Competency was also taken into consideration, prompted by the research team’s suggestion and recurring issues raised during interviews, in response to the potential challenge of insufficient competence within our specific context. So overall, In this study, we assessed stakeholders using five factors, which we defined as follows. The broad definition of power is the ability of stakeholders to influence policy or program implementation. We break down this broad definition into its dimensions using the following criteria: power, influence, and competency. This study defines “power” as the amount of resources a stakeholder possesses and their capacity to mobilize them. Influence is defined as a stakeholder’s ability to exert power over other stakeholders. Competency refers to the technical and professional skills and knowledge required for a stakeholder to fulfill their role. “Position” relates to a stakeholder’s stance on a specific policy, which can range from active support to active opposition, with varying degrees of neutrality in between [21]. “Interest” represents a stakeholder’s motivation for the policy [19]. We define a stakeholder connection as an actual channel for transmitting messages from one stakeholder to another.

### Rigor and trustworthiness

To enhance the rigor and trustworthiness of our findings, we followed the Guba and Lincoln approach, which involved considering criteria such as credibility, confirmability, dependability, transferability, and authenticity [22, 23]. To address these criteria, we implemented various strategies throughout the study, including peer debriefing to provide an external check on the research process for credibility, involving multiple authors and gathering a list of existing stakeholders from multiple data sources (Searching relevant documents, laws, regulations), using theoretical framework to gather a comprehensive list of stakeholders (modified tool) of the for dependability, utilizing maximal variation sampling for transferability, member checking by contributors for confirmability, and incorporating citations from nearly all individuals for authenticity.

### Social network analysis

SNA was used to analyze connections among stakeholders in this study [24]. The fundamental concept underlying SNA is that network connections and their structure are significant and can be independently analyzed, irrespective of individual stakeholder characteristics [25]. Centrality measures, which reveal the structural importance of a stakeholder within a network, are the most frequently employed metrics in SNA. We employed degree, betweenness, and closeness centrality measures (see Supplementary Table 3). Furthermore, we utilized the modularity index to partition the network into clusters of smaller networks based on their structural attributes [26].

We used Stata software (Version 17, Stata Corporation, College Station, Texas, USA) and Microsoft Excel (2016) for statistical analysis and creating figures. Additionally, we employed the networkx package in Python [27] and Gephi for SNA.

## Results

We identified 74 stakeholders through interviews and ranked them based on five parameters. The final list of the most important stakeholders consists of 23 organizations or groups involved in decision-making and policy development related to evidence-based TPM in Iran (refer to Table 1).

**Table 1:** Power, position, interest, influence, and competency of stakeholders

Label	Power^*^	Competency	Position^**^	Interest	Influence
CP.	Medium	Medium	S	Medium-High	Medium
CAMIC	Low	Medium	N	Medium	Low
GCTM-WHO	Low-Medium	High	S	High	Low-Medium
Guilds	Medium	Low-Medium	O	High	Low-Medium
IAMS	Low-Medium	High	S	High	High
Insurances	Medium-High	Low-Medium	N	Medium	Medium
IRIB	Low-Medium	Low-Medium	N	Medium	Low-Medium
IRMC	Medium	Medium	S	High	Medium
judicial	Medium-High	Medium	N	Low	Medium
Maraji	Low-Medium	Medium	S	Medium	Medium
MOHME	High	Medium	S	High	Medium
Parliament	Low-Medium	Low-Medium	N	Low-Medium	Medium-High
PhC	Medium-High	Medium-High	O	Medium	Medium
Public	High	Low-Medium	N	Low-Medium	Medium
QTC	Low-Medium	Low-Medium	O	Medium-High	Low
Quacks	Low-Medium	Low	O	High	Low-Medium
S&C	Medium	Low-Medium	O	Medium-High	Medium
SAMT	Low-Medium	Medium	N	Low-Medium	Medium-High
SCCR	Low-Medium	Medium-High	N	Medium-High	Medium-High
SGB	Medium	Medium-High	N	Medium	High
TPMRC	Low	Low-Medium	S	High	Low-Medium
TPMS	Medium	Low-Medium	S	High	Low-Medium
VPST	Medium	Medium	N	Medium-High	Low-Medium
MOHME subordinates					
DTPM	Medium	Low-Medium	S	High	Low-Medium
DTPPh	Medium	Medium	S	High	Low-Medium
IFDA	Medium-High	Medium	S	High	Medium
OTPM	Medium-High	Low-Medium	S	High	Low-Medium
UMS	High	Low-Medium	S	High	Medium
VCE	Low-Medium	Low-Medium	S	Low-Medium	Low-Medium
VCH	Low-Medium	Low-Medium	N	Low-Medium	Low-Medium
VCRT	Medium	Medium	S	High	Low-Medium
VCT	Medium-High	Low-Medium	S	Medium	Medium

*High = 8 -10, Medium-High = 6-7.9, Medium= 4-5.9, Low-Medium=2-3.9, Low=0-1.9

**O = Opponent (Below 4), N= Neutral (4.1-5.9), S = Supporter (above 6)

The highest power reported was the Ministry of Health and Medical Education (MOHME) and the public. The supreme governing bodies (SGB), judicial and enforcement system (Judicial), pharmaceutical companies (PhC), and insurance companies were considered middle to high-power stakeholders in promoting evidence-based practice (EBP) of TPM.

Our analysis found SGB and the Iranian Academy of Medical Sciences (IAMS) to have the strongest influence on other stakeholders, with IAMS being the only highly influential and interested stakeholder competent in promoting EBP of TPM. WHO and IAMS had the highest competency, followed by PhC, SGB, and the Supreme Council of the Cultural Revolution (SCCR), reported as a medium to a highly competent organization for promoting EBP of TPM (Table 1). Roughly 20% of stakeholders were opponents to promoting EBP in TPM. Public, insurance, and non-health-related governmental organizations such as SGB, SCCR, parliament, Vice Presidency for Science and Technology (VPST), judicial, Ministry of Industry, Mine and Trade (SAMT), The Islamic Republic of Iran Broadcasting (IRIB), Insurances, Public, and other international centers active in complementary medicine were neutral to this policy. The lowest interest was in the Judicial, SAMT, parliament, and public. Most stakeholders within the MOHME subdivision were supportive of promoting EBP in TPM, except for the Vice-Chancellery for Health. The universities of medical sciences had the highest power and influence, while the overall competency of the MOHME subdivision was medium or lower (Table 1).

**Fig. 1:**
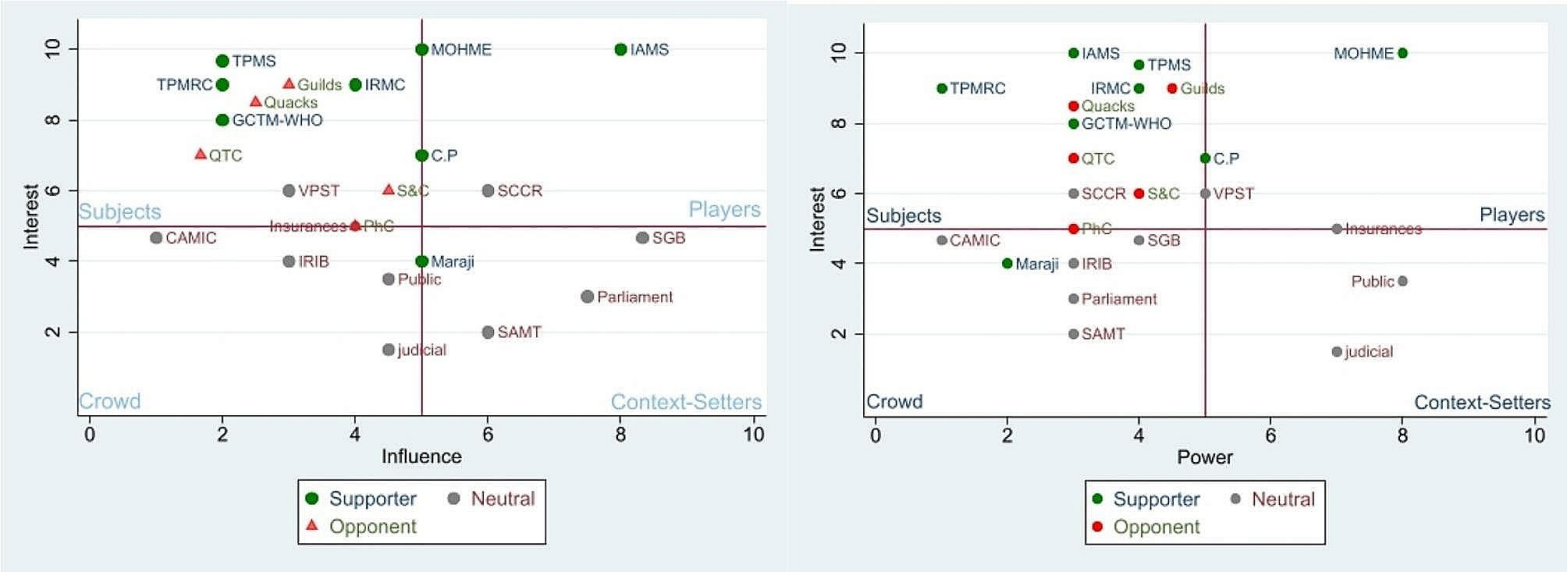
**A** - Interest/Influence, **B** - Power/Position, each consists of four quadrants of players (IAMS, SCCR, MOHME), context setters (SGB, Parliament, SAMT, the public, judiciary), and several stakeholders in subjects and the crowd

The highest mean of influence (4.78) and power (4.4) and the lowest mean of interest (4.03) were found in stakeholders with neutral positions. Stakeholders who supported the policy of EBM in TPM had the highest interest (8.33) (Table 2). MOHME was a highly interested and powerful policy supporter, and IAMS was the most influential supporter (Fig. 1).

**Table 2:** Sum and means of interest, influence, competency, power over position

	Sum (CI)	Mean (CI)
Interest		
Supporter	66.67 (54.75–78.58)	8.33 (6.84–9.82)
Neutral	40.34 (30.23–50.44)	4.03 (3.02–5.04)
Opponent	35.5 (27.74–43.25)	7.1 (5.54–8.65)
Influence		
Supporter	33 (20.68–45.31)	4.12 (2.58–5.66)
Neutral	47.83 (33.26–62.39)	4.78 (3.32–6.23)
Opponent	15.67 (10.39–20.94)	3.13 (2.07–4.18)
Competency		
Supporter	36.67 (23.54–49.7)	4.58 (2.94–6.22)
Neutral	42.3 (31.1-53.58)	4.23 (3.10–5.35)
Opponent	17.75 (8.03–27.46)	3.55 (1.60–5.49)
Power		
Supporter	30 (17.55–42.44)	3.75 (2.19–5.30)
Neutral	44 (29.10-58.89)	4.4 (2.91–5.88)
Opponent	17.5 (14.22–20.77)	3.5 (2.84–4.15)

### Social network analysis

The network had 23 nodes and 208 edges, with an overall density of 0.411 and an average shortest path of 1.947. SBG (0.379), MOHME (0.303), IAMS (0.288), and Maraji (0.273) exhibited the highest indegree centrality, while SGB (0.424), MOHME (0.348), Maraji (0.303), and PhC (0.303) displayed the highest outdegree centrality. IRIB (0.611), MOHME (0.595), PhC (0.595), and Quacks (0.595) showed the highest closeness centrality. The betweenness centrality of PhC, IAMS, TPMS, and VPST was 0.115, 0.111, 0.07, and 0.067, respectively (Table 3).

**Table 3:** Network parameters of stakeholders of evidence-based practice of traditional persian medicine

Label	Outdegree	Degree	Closeness Centrality	Betweenness centrality
CP	0.197	0.182	0.512	0.011
CAMIC	0.061	0.076	0.4	0.007
GCTM-WHO	0.045	0.076	0.379	0.004
Guilds	0.121	0.045	0.524	0.046
IAMS	0.152	0.288	0.579	0.111
Insurances	0.152	0.242	0.537	0.012
IRIB	0.242	0.258	0.611	0.018
IRMC	0.152	0.167	0.564	0.03
judicial	0.258	0.227	0.55	0.027
Maraji	0.303	0.273	0.579	0.057
MOHME	0.348	0.303	0.595	0.064
Parliament	0.212	0.258	0.579	0.024
PhC	0.303	0.197	0.595	0.115
Public	0.212	0.167	0.5	0.066
QTC	0.136	0.152	0.512	0.02
Quacks	0.197	0.273	0.595	0.043
S&C	0.273	0.182	0.478	0.029
SAMT	0.015	0.152	0.355	0.031
SCCR	0.227	0.182	0.478	0.023
SGB	0.424	0.379	0.537	0.047
TPMRC	0.136	0.167	0.489	0.022
TPMS	0.258	0.212	0.55	0.07
VPST	0.121	0.091	0.564	0.067

**Fig. 2:**
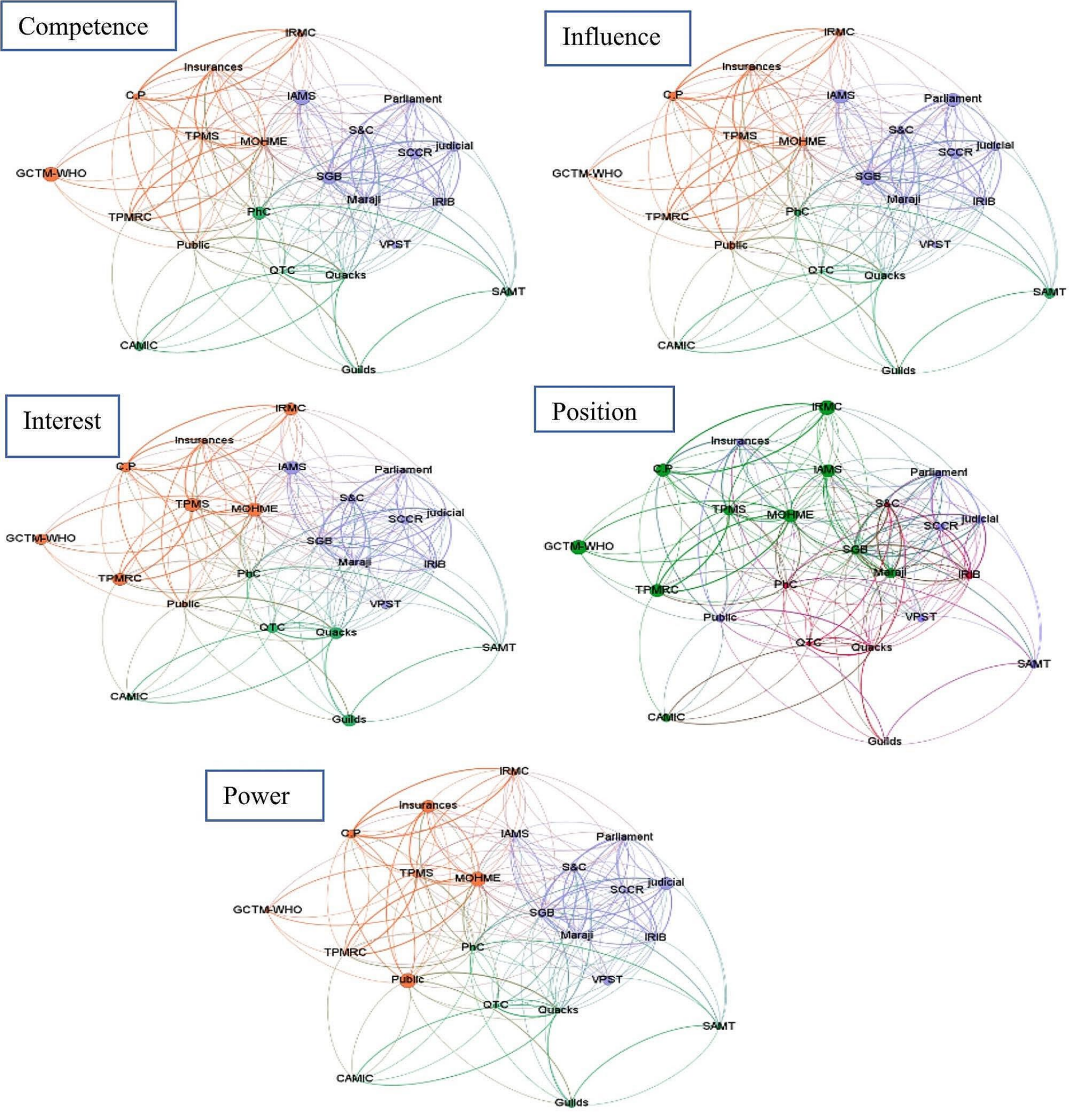
Visualization of the network of stakeholders: The size of nodes in each figure suggests the stakeholder’s five factors (Power, Interest, Influence, Position, Competency); the color of each stakeholder corresponds to the cluster to which it belongs

Three clusters of stakeholders were identified in a network analysis using a modularity algorithm, each represented by distinct colors in the network figures (see Supplementary Table 4). The modularity score for this network was 0.217. The stakeholders in the first cluster (modularity class = 0) were supporters, with a score of 7.25 for the policy. The second (modularity class = 1) and third (modularity class = 2) clusters were characterized as neutral, with a score of 5.29, and opponents, with a score of 3.44, respectively (see Table 4; Fig. 2).

**Table 4:** The mean value of stakeholder features in different clusters of the network found by the modularity index

Modularity class	Position(SD)	Power(SD)	Competency(SD)	Interest(SD)	Influence(SD)
0	7.25 (6.02–8.47)	5 (3.16–6.83)	3.70 (2.29–5.12)	7.64 (5.93–9.35)	3.56 (2.57–4.55)
1	5.29 (4.41–6.17)	3.77 (2.754.80)	4.77 (3.41–6.13)	5.01 (3.35–6.68)	5.53 (4.12–6.94)
2	3.44 (2.03–4.85)	2.91 (1.97–3.86)	4 (2.30–5.71)	6.02 (3.78–8.26)	3.02 (1.51–4.54)

## Discussion

This study aimed to identify stakeholders involved in implementing the policy of EBM in TPM and evaluate their power, influence, interest, position, and competency. The analysis revealed that AIMS and MOHME are influential and powerful supporting players who should be given priority for engagement and communication. SNA showed that the network density among the stakeholders was low, indicating a lack of collaboration and connection among the key stakeholders. The stakeholders can be categorized into three subcommunities based on their positions as supporters, neutrals, and opponents. This study highlighted the roles of stakeholders in the network. It demonstrated that AIMS could bridge the structural gap between supporter and neutral clusters. Key connectors or bottlenecks were identified as Phc, while the Public, SGB, and guilds acted as gatekeepers or brokers in the network. Overall, this study provides valuable insights into the challenges and opportunities for promoting EBP in TPM in Iran.

### Classical stakeholder analysis

Two primary supporting stakeholders were AIMS and MOHME, both of which held significant influence and power, respectively. The classical stakeholder analysis has revealed that stakeholders falling into the “player” category (influential, powerful, and interested) should receive the highest priority for engagement and communication, as they possess the significant potential to impact the project’s success or decisions. In our study, we did not identify any key opposing stakeholders, which may be attributed to the disorganized and individually oriented nature of the opposing network, a lack of transparency among opponents, or the inability to access information related to the opposing network.

Our study found that many powerful and influential stakeholders, such as the SGB, Judiciary, Parliament, the public, and insurance providers, were either neutral or uninterested in supporting the policy of EBM in TPM. Other published stakeholder analyses related to health policies in Iran also identify the Parliament, MOHME, the Judiciary, insurance providers, and UMS as powerful or influential stakeholders [28–31]. The unwillingness of powerful and influential stakeholders to participate is a major challenge, which has also been demonstrated in other stakeholder analyses in Iran [28, 29]. These mentioned stakeholders, often referred to as ‘Context-setters’ (influential or powerful but not interested), should be informed and engaged, as their decisions and actions can significantly impact the project or decision. We found that the public is a powerful stakeholder; this result contrasts with some other stakeholder analyses, which considered the public as a less powerful, passive stakeholder [28, 29]. We believe that the public’s power stems from their financial resources, driven by the widespread need for and usage of TPM services [7, 11, 13].

Our findings suggest that the MOHME held significant influence, displayed interest, and provided support for this policy. MOHME possesses both structural and human resources related to TPM. These resources include institutions such as the Office of Traditional Persian Medicine (OTPM), several Traditional Persian Medicine Research Centers (TPMRC), and Traditional Persian Medicine Specialists (TPMS), many of whom have medical backgrounds. Given the presence of these resources within MOHME, it appears that this stakeholder may not have taken substantial action and has not prioritized this policy. Consider that the highest level of competency among supporters was identified in WHO-GCTIM as an international stakeholder and AIMS as a national organization. Our study also revealed another existing challenge: key supporters like MOHME, IRMC, CP, TPMS, and TPMRC exhibit medium to low levels of competency. Competency levels for MOHME’s subordinates were even lower.

### Social network analysis

The network’s density was relatively low when compared to other stakeholder networks in health-related policy in Iran [28]. This suggests that critical stakeholders in this policy were not well connected. The density of the second cluster (the neutral cluster) appears to be the highest in the network. Therefore, the density in the clusters of supporters and opponents is lower than the overall estimated value. Another challenge in the network of supporters for this policy is the lack of connections and collaboration among key stakeholders in this field. We identified three clusters of stakeholders in this network, which we can categorize as highly influential, uninterested, and neutral stakeholders. The network structure of these stakeholders supports the findings from classical stakeholder analysis. We propose naming these clusters as supporters, neutrals, and opponents based on their positions.

In a social network, a structural hole is a gap where two contacts or groups are not directly connected, and brokers can bridge this gap by connecting them [32]. We found significant structural holes that could impact collaboration and the policy process [33]. It appears that AIMS could bridge the structural gap between the supporter and neutral clusters. There is a lack of a stronger connection between AIMS and IRMC, MOHME and IRMC, AIMS and Parliament, AIMS and S&C, and TPMS and CP, as well as between international stakeholders (GCTM-WHO and CAMIC) and TPMRC. Although studies have shown that increasing connections between nodes (reducing structural holes) reduces flexibility and innovation, reducing structural holes can lead to decreased cooperation [33, 34]. It seems that trying to establish these connections and relying on AIMS and the public brokerage role can help advance policy.

The SNA reveals that PhC was a node with high betweenness and closeness centrality; these nodes are often referred to as key connectors, bottlenecks, or hubs of the network [35, 36]. These nodes can control the flow of information and resources within a network. The IRIB also had high closeness but relatively low betweenness centrality, indicating that this stakeholder was essential for local communication within a specific cluster in the network. Other stakeholders, such as Parliament, IRMC, and Quacks, can be considered local communicators within their respective clusters. The Public, SGB, and guilds had relatively high betweenness and relatively low closeness centrality; these nodes are often known as gatekeepers or brokers in a network. These nodes serve as critical connectors between different parts of the network, and their removal could result in network fragmentation [35, 36]. The Public, being the target population and final user of the service, plays a role in connecting different parts of the network to each other. International stakeholders (CAMIC and GCTM-WHO) were found to be peripheral nodes due to their low centrality measures. CAMIC includes institutions such as the Ministry of Ayush in India, Hamdard Universities, and other Unani medicine organizations. Overall, MOHME and Maraji were identified as high-ranking stakeholders based on four different centrality measures (**Supplementary Table 5**).

### What should we do?

Based on the results of the stakeholder analysis, here are some recommendations for promoting EBP in the field of TPM:

Enhancing Competency: A deficiency in competency has been identified in various health-related stakeholders, including MOHME, IRMC, CP, TPMS, and TPMRC. To advance the adoption of EBP within the field of TPM, it is imperative to develop a competency map for each key stakeholder. Furthermore, training interventions and awareness campaigns should be initiated to augment their knowledge, attitudes, and implementation of EBP. Achieving this goal necessitates the cultivation of shared competencies among all healthcare professionals. Specifically, we recommend that MOHME bolster its structural and human resources through this process, thereby fostering the creation of reliable evidence to support TPM practices.

Address managerial competencies: Stakeholders related to managerial competencies, such as leadership, change management, and financial management, require more attention. This attention will impact not only this policy but any program or policy. Therefore, addressing these managerial competencies is essential to promote the EBP of TPM.

Encourage connections and collaboration: The absence of connections and collaboration among key stakeholders in the realm of TPM policies has also been identified as a weakness. Promoting connections and collaboration among these stakeholders, including MOHME, IAMS, CP, IRMC, and international partners, can assist in establishing a more robust network of supporters and advancing EBP.

Persuade powerful and influential stakeholders and advocate for policy with significant stakeholders: The study revealed that numerous powerful and influential stakeholders, as well as key connectors in the network, maintain a neutral position regarding this policy. Therefore, it presents an opportunity for proponents of this policy to persuade these stakeholders, including AIMS, the public, MOHME, insurance, the judiciary, Parliament, SGB, and universities of medical sciences (UMS).

Addressing opposition and threats: The opposition from religious institutions and clergy to this policy poses a significant threat that must be dealt with. Administrative corruption has also been reported as a challenge in Iran [37, 38], and PhC may oppose the policy due to their financial interests. Therefore, it is essential to address these threats to promote the EBP of TPM.

Utilize strengths: MOHME has been recognized as a powerful, interested, and supportive stakeholder for this policy. Leveraging MOHME’s strengths, including the OTPM, numerous TPMRCs, and TPMS, can significantly contribute to the promotion of EBP within TPM.

Seek Assistance and Form Alliances: Seek assistance from influential and powerful stakeholders, such as AIM and the public, to act as intermediaries in advancing the policy. Additionally, establish relations with GCTM-WHO (a peripheral node) to leverage their expertise and maintain engagement with local communicators.

It seems that MOHME should revise the regulatory policies of IFDA for herbal drugs to encourage the PhC to invest exclusively in scientifically proven effective herbal medicines. We also recommend that MOHME and its allies promote this policy to influential, powerful, neutral, and less interested stakeholders. Another aspect of the policy should center on legally combating quackery through the influence and power of IRMC, the judicial system, and the enforcement system.

### Limitations

Accessing the opponents’ and quackery network was a complex task, and we were unable to completely grasp their perspective in this study. Therefore, we recommend a more focused investigation of the quackery network. Stakeholder positions may evolve over time, as evidenced by recent developments involving the SCCR’s secretary, underscoring the time-dependent nature of cross-sectional stakeholder analysis.

## Conclusion

This study reveals the presence of several influential and disinterested stakeholders within the network. The support network presents favorable opportunities as well as certain challenges for policy implementation. To tackle these challenges, various actions can be taken, such as advocating for the policy to uninterested yet significant nodes in the network, improving competence through educational interventions, strengthening international relations, and harnessing existing strengths.

### Electronic supplementary material

Below is the link to the electronic supplementary material.

Supplementary Material 1

## Data Availability

All information required is given in the text and supplementary materials, other supplementary information can be obtained upon email from the corresponding author.

## Abbreviations

CAMIC: Other international centers active in complementary medicine
CP: Doctors of conventional medicine
DTPM: Traditional Persian medicine schools and departments
DTPPh: Traditional pharmacy department
EBM: Evidence-based medicine
EBP: Evidence-based practice
GCTM-WHO: Global Centre for Traditional Medicine - World Health Organization
Guilds: Herbal remedy store & Massage
IAMS: Iranian Academy of Medical Sciences
IFDA: Iranian Food and Drug Administration
IRIB: The Islamic Republic of Iran Broadcasting
IRMC: Medical Council of the Islamic Republic of Iran
Judicial: Judicial & Enforcement system
Maraji: Religious References
MOHME: Ministry of Health and Medical Education
OTPM: Office of Traditional Persian Medicine
Parliament: Islamic Consultative Assembly
PhC: Pharmaceutical companies
QTC: Quackery education center
Quacks: Non-medical quack and therapist
S&C: Seminary and clergy
SAMT: Ministry of Industry, Mine and Trade
SCCR: Supreme Council of the Cultural Revolution
SGB: Supreme governing bodies
SNA: Social network analysis
TPM: Traditional Persian medicine
TPMRC: Iranian traditional medicine and traditional pharmaceutical research center
TPMS: Iranian traditional medicine doctors
UMS: Universities of medical sciences
VCRT: Vice President of Research and Technology of MOHME
VCE: Vice President of Education of MOHME
VCH: Vice President of Health of MOHME
VCT: Vice President of Treatment of MOHME
VPST: Vice-Presidency for Science and Technology

## References

[1] World Health Organization. WHO global report on traditional and complementary medicine 2019. Geneva: World Health Organization; 2019 2019.

[2] Sackett DL (1997). Evidence-based medicine. Semin Perinatol.

[3] Mozafarpour S, Sadeghizadeh A, Kabiri P, Taheri H, Attaei M, Khalighinezhad N (2011). Evidence-based medical practice in developing countries: the case study of Iran. J Eval Clin Pract.

[4] Ghojazadeh M, Azami-Aghdash S, Pournaghi Azar F, Fardid M, Mohseni M, Tahamtani T (2015). A systematic review on barriers, facilities, knowledge and attitude toward evidence-based medicine in Iran. J Anal Res Clin Med.

[5] Moosavi A, Sadeghpour A, Azami-Aghdash S, Derakhshani N, Mohseni M, Jafarzadeh D (2020). Evidence-based medicine among health-care workers in hospitals in Iran: a nationwide survey. J Educ Health Promotion.

[6] Zargaran A. Reviewing six Health System building blocks of Traditional and Complementary Medicine in Iran. 2019.

[7] Moeini R, Mozaffarpur SA, Mojahedi M, Nasrolahpour Shirvani SD, Gorji N, Saghebi R (2021). The prevalence of complementary and alternative medicine use in the general population of Babol, North of Iran, 2018. BMC Complement Med Ther.

[8] Hashempur MH, Heydari M, Mosavat SH, Heydari ST, Shams M (2015). Complementary and alternative medicine use in Iranian patients with diabetes mellitus. J Integr Med.

[9] Behnood-Rod A, Afzali Poor Khoshkbejari M, Pourzargar P, Hassanzadeh M, Moharamzad Y, Foroughi F (2018). Complementary and alternative medicine use among Iranian patients attending urban outpatient general practices. Complement Ther Clin Pract.

[10] Amirmoezi F, Araghizadeh M, Mohebbinia Z, Kamfiroozi R, Haghpanah S, Bordbar M (2017). Use of complementary and alternative Medicine among Iranian Cancer patients in South of Iran. Int J Cancer Manag.

[11] Ghaedi F, Dehghan M, Salari M, Sheikhrabori A (2017). Complementary and alternative Medicines: usage and its determinant factors among outpatients in Southeast of Iran. J Evid Based Complement Altern Med.

[12] Hajigholami A, Moazam E, Salehi M, Ansari H (2022). Complementary and alternative medicine in Cancer patients and the causes of Tendency to Use such treatments in Isfahan, Iran. Adv Biomedical Res.

[13] Jafari A, Zanganeh M, Kazemi Z, Lael-Monfared E, Tehrani H (2021). Iranian healthcare professionals’ knowledge, attitudes, and use of complementary and alternative medicine: a cross sectional study. BMC Complement Med Ther.

[14] Kolangi F, Memariani Z, Bozorgi M, Mozaffarpur SA, Mirzapour M (2018). Herbs with potential nephrotoxic effects according to the traditional persian medicine: review and assessment of scientific evidence. Curr Drug Metab.

[15] Joharchi MR, Amiri MS (2012). Taxonomic evaluation of misidentification of crude herbal drugs marketed in Iran. Avicenna J Phytomedicine.

[16] Pasalar M, Daneshfard B, Lankarani KB (2020). Complementary and alternative medicine-related drug-induced Liver Injury in Iran. J Clin Translational Hepatol.

[17] Brugha R, Varvasovszky Z (2000). Stakeholder analysis: a review. Health Policy Plann.

[18] World Health Organization. Monitoring the building blocks of health systems: a handbook of indicators and their measurement strategies. World Health Organization; 2010.

[19] Schmeer K. Stakeholder analysis guidelines. Policy toolkit for strengthening health sector reform. 1999;1:1–35.

[20] Varvasovszky Z, Brugha R (2000). A stakeholder analysis. Health Policy Plann.

[21] Balane MA, Palafox B, Palileo-Villanueva LM, McKee M, Balabanova D (2020). Enhancing the use of stakeholder analysis for policy implementation research: towards a novel framing and operationalised measures. BMJ Global Health.

[22] Nowell LS, Norris JM, White DE, Moules NJ (2017). Thematic analysis: striving to meet the trustworthiness criteria. Int J Qualitative Methods.

[23] Kyngäs H, Kääriäinen M, Elo S, Kyngäs H, Mikkonen K, Kääriäinen M (2020). The trustworthiness of Content Analysis. The application of content analysis in Nursing Science Research.

[24] Reed MS, Graves A, Dandy N, Posthumus H, Hubacek K, Morris J (2009). Who’s in and why? A typology of stakeholder analysis methods for natural resource management. J Environ Manage.

[25] Borgatti SP, Mehra A, Brass DJ, Labianca G (2009). Network analysis in the social sciences. Science.

[26] Blondel VD, Guillaume J-L, Lambiotte R, Lefebvre E (2008). Fast unfolding of communities in large networks. J Stat Mech: Theory Exp.

[27] Hagberg A, Swart P, Chult S. D. Exploring network structure, dynamics, and function using NetworkX. Los Alamos National Lab.(LANL), ;; 2008. Los Alamos, NM (United States).

[28] Shahabi S, Ahmadi Teymourlouy A, Shabaninejad H, Kamali M, Lankarani KB (2020). Financing of physical rehabilitation services in Iran: a stakeholder and social network analysis. BMC Health Serv Res.

[29] Atashbahar O, Sari AA, Takian A, Olyaeemanesh A, Mohamadi E, Barakati SH (2021). Integrated early childhood development policy in Iran: a stakeholder analysis. BMC Health Serv Res.

[30] Heydari M, Seyedin H, Jafari M, Dehnavieh R (2018). Stakeholder analysis of Iran’s health insurance system. J Educ Health Promotion.

[31] Olyaaeemanesh A, Jaafaripooyan E, Abdollahiasl A, Davari M, Mousavi SM, Delpasand M (2021). Pharmaceutical subsidy policy in Iran: a qualitative stakeholder analysis. Health Res Policy Syst.

[32] Lin Z, Zhang Y, Gong Q, Chen Y, Oksanen A, Ding AY (2022). Structural hole theory in Social Network Analysis: a review. IEEE Trans Comput Social Syst.

[33] Gargiulo M, Benassi M (2000). Trapped in your own net? Network cohesion, structural holes, and the adaptation of social capital. Organ Sci.

[34] Ahuja G, Collaboration, Networks (2000). Structural holes, and Innovation: a longitudinal study. Adm Sci Q.

[35] Chou B-H, Suzuki E, editors. Discovering community-oriented roles of nodes in a Social Network. Data Warehousing and Knowledge Discovery; 2010 2010//; Berlin, Heidelberg: Springer Berlin Heidelberg.

[36] Wasserman S, Faust K. Social network analysis: Methods and applications. 1994.

[37] Firouzjaeian AA, Mahmoudian M (2022). Systemic review of Scientific studies on administrative corruption in Iran. Half -Yearly Social Probl Iran.

[38] Joulaei H, Bagheri Lankarani K, Shahabi S, Azizmohammadi F, Keshavarzian A. Critical Analysis of Corruption in Iran’s Health Care System and Its Control Strategies. 2021;23(3):e115669.

