# Supplementary material for: Evidence-based practice in traditional persian medicine (TPM): a stakeholder and social network analysis
Source: BMC Complement Med Ther. 2024 Jul 3;24:253. doi: 10.1186/s12906-024-04564-5 (PMC11223371; doi:10.1186/s12906-024-04564-5)
Supplement: Supplementary file 1 — Supplementary Material 1 [file 12906_2024_4564_MOESM1_ESM.docx]

| Supplementary Table 1: list the interviewee, some persons included in several specialties; therefore, the total number is more than 24 | |
| --- | --- |
| Specialty | **Number** |
| Traditional Persian medicine specialist | 12 |
| Conventional medicine physicians | 5 |
| Pharmacist | 3 |
| Politicians | 3 |
| Health policymaker | 2 |
| Other | 3 |

| Supplementary Table 2: A modified version of the six building blocks framework used for the semi-structured interview, the participants were asked, "Is there another stakeholder who plays a key role in the process of policymaking or implementation in different aspects of TPM practice at different levels?” | | | | | | | | | | |
| --- | --- | --- | --- | --- | --- | --- | --- | --- | --- | --- |
| Aspect      Level | | | Financing | The field of policy and planning | Service delivery | ​​Supervision and regulatory | Knowledge generation and information | Education field (to the public) | Supplying human resources | Drug and device production and supply chain |
| National | State | Government |  |  |  |  |  |  |  |  |
|  |  | Parliament |  |  |  |  |  |  |  |  |
|  |  | Judiciary |  |  |  |  |  |  |  |  |
|  |  | other |  |  |  |  |  |  |  |  |
|  | non-State |  |  |  |  |  |  |  |  |  |
| International |  |  |  |  |  |  |  |  |  |  |

| Supplementary Table 3. An illustration of network measures in an abstract network | | | | | |
| --- | --- | --- | --- | --- | --- |
| Measure | | Definition | | Measures value | 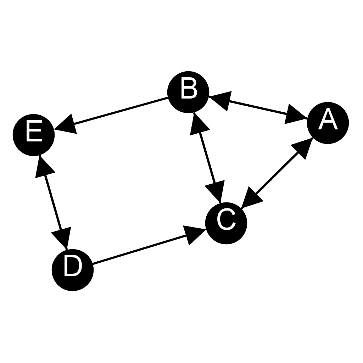 |
| Density | Density measures the proportion of existing ties in a network compared to the total number of possible ties.  $\frac{Total Connections}{Total possible connections}$  The network on the right-hand side has ten links/edges (considering directional links). The possible number of ties is 5×4 = 20. Hence, density equals 10÷20 = 0.5 | | | 0.5 |  |
| Indegree Centrality | For a node in a network with size N, the indegree is  $\frac{Total number of incoming edges}{N-1}$ | | | $A=\frac{2}{5-1}=0.5$  $B=\frac{2}{5-1}=0.5$  $C=\frac{3}{5-1}=0.75$  $D=\frac{1}{5-1}=0.25$  $E=\frac{2}{5-1}=0.5$ |  |
| Outdegree Centrality | | | -  For a node in a network with size N, the outdegree is  $\frac{Total number of outgoing edges}{N-1}$ | $A=\frac{2}{5-1}=0.5$  $B=\frac{3}{5-1}=0.75$  $C=\frac{2}{5-1}=0.5$  $D=\frac{2}{5-1}=0.5$  $E=\frac{1}{5-1}=0.25$ |  |
| Closeness Centrality | | | It measures how close a node is to all other nodes in the network. The closeness centrality of a node in a network with size N is defined as the inverse of the sum of the shortest path distances between that node and all other nodes in the network. $\frac{N-1}{\sum_{1}^{N} shortest path distances from that node}$ | $A=\frac{5-1}{1+1+2+3}=0.571$  $B=\frac{5-1}{1+1+1+2}=0.8$  $C=\frac{5-1}{1+1+2+3}=0.571$  $D=\frac{5-1}{1+1+2+2}=0.667$  $E=\frac{5-1}{1+2+3+3}=0.44$ |  |
| Betweenness centrality | | | It measures how often a node appears on the shortest path between other pairs of nodes in the network. The betweenness centrality of a node is defined as the number of shortest paths between all pairs of nodes in the network that pass through that node.  For node A  The betweenness centrality for node A is 0 since this node does not fall in the shortest path of any other node pairs.  For node D  The right-hand network has two shortest paths between nodes C and E; one has node D (CDE). The other (CBE) does not have node D. Hence, node D can claim only 0.5 (out of 1) for the shortest path between nodes C and E. The other shortest path in the network is ABE, which does not contain node D. Thus, the betweenness centrality for node D is  $D=\frac{0.5}{1+1}=0.25$ | A=0  $B$=033  $C$=033  $D=0.25$  $E=$0.25 |  |

| Supplementary Table 4: Results of different clustering algorithms both show relatively similar communities. | | |
| --- | --- | --- |
| Label | Cluster_Louvian algorithm | Cluster_leiden algorithm |
| IAMS | 1 | 0 |
| MOHME | 0 | 0 |
| SGB | 1 | 1 |
| IRMC | 0 | 0 |
| SCCR | 1 | 1 |
| PhC | 2 | 2 |
| TPMS | 0 | 0 |
| Insurances | 0 | 0 |
| Maraji | 1 | 1 |
| Parliament | 1 | 1 |
| GCTM-WHO | 0 | 0 |
| C.P | 0 | 0 |
| Public | 0 | 0 |
| VPST | 1 | 1 |
| TPMRC | 0 | 0 |
| IRIB | 1 | 1 |
| judicial | 1 | 1 |
| Quacks | 2 | 2 |
| S&C | 1 | 1 |
| SAMT | 2 | 2 |
| Guilds | 2 | 2 |
| CAMIC | 2 | 2 |
| QTC | 2 | 2 |

| **Supplementary Table 5:** Top stakeholders based four different centrality meausres | | | | |
| --- | --- | --- | --- | --- |
| Node-Id | betweennessCentrality | closenessCentrality | inDegreeCentrality | outDegreeCentrality |
| PhC | 0.115 | 0.595 | 0.197 | 0.303 |
| IAMS | 0.111 | 0.579 | 0.288 | 0.152 |
| TPMS | 0.07 | 0.55 | 0.212 | 0.258 |
| VPST | 0.067 | 0.564 | 0.091 | 0.121 |
| Public | 0.066 | 0.5 | 0.167 | 0.212 |
| MOHME | 0.064 | 0.595 | 0.303 | 0.348 |
| Maraji | 0.057 | 0.579 | 0.273 | 0.303 |
| SGB | 0.047 | 0.537 | 0.379 | 0.424 |
| Guilds | 0.046 | 0.524 | 0.045 | 0.121 |
| Quacks | 0.043 | 0.595 | 0.273 | 0.197 |
| SAMT | 0.031 | 0.355 | 0.152 | 0.015 |
| IRMC | 0.03 | 0.564 | 0.167 | 0.152 |
| S&C | 0.029 | 0.478 | 0.182 | 0.273 |
| judicial | 0.027 | 0.55 | 0.227 | 0.258 |
| Parliament | 0.024 | 0.579 | 0.258 | 0.212 |
| SCCR | 0.023 | 0.478 | 0.182 | 0.227 |
| TPMRC | 0.022 | 0.489 | 0.167 | 0.136 |
| QTC | 0.02 | 0.512 | 0.152 | 0.136 |
| IRIB | 0.018 | 0.611 | 0.258 | 0.242 |
| Insurances | 0.012 | 0.537 | 0.242 | 0.152 |
| C.P | 0.011 | 0.512 | 0.182 | 0.197 |
| CAMIC | 0.007 | 0.4 | 0.076 | 0.061 |
| GCTM-WHO | 0.004 | 0.379 | 0.076 | 0.045 |

**Supplementary Figure 1:** Stakeholder network considering all stakeholders. The size of a node is proportional to its degree centrality value.**
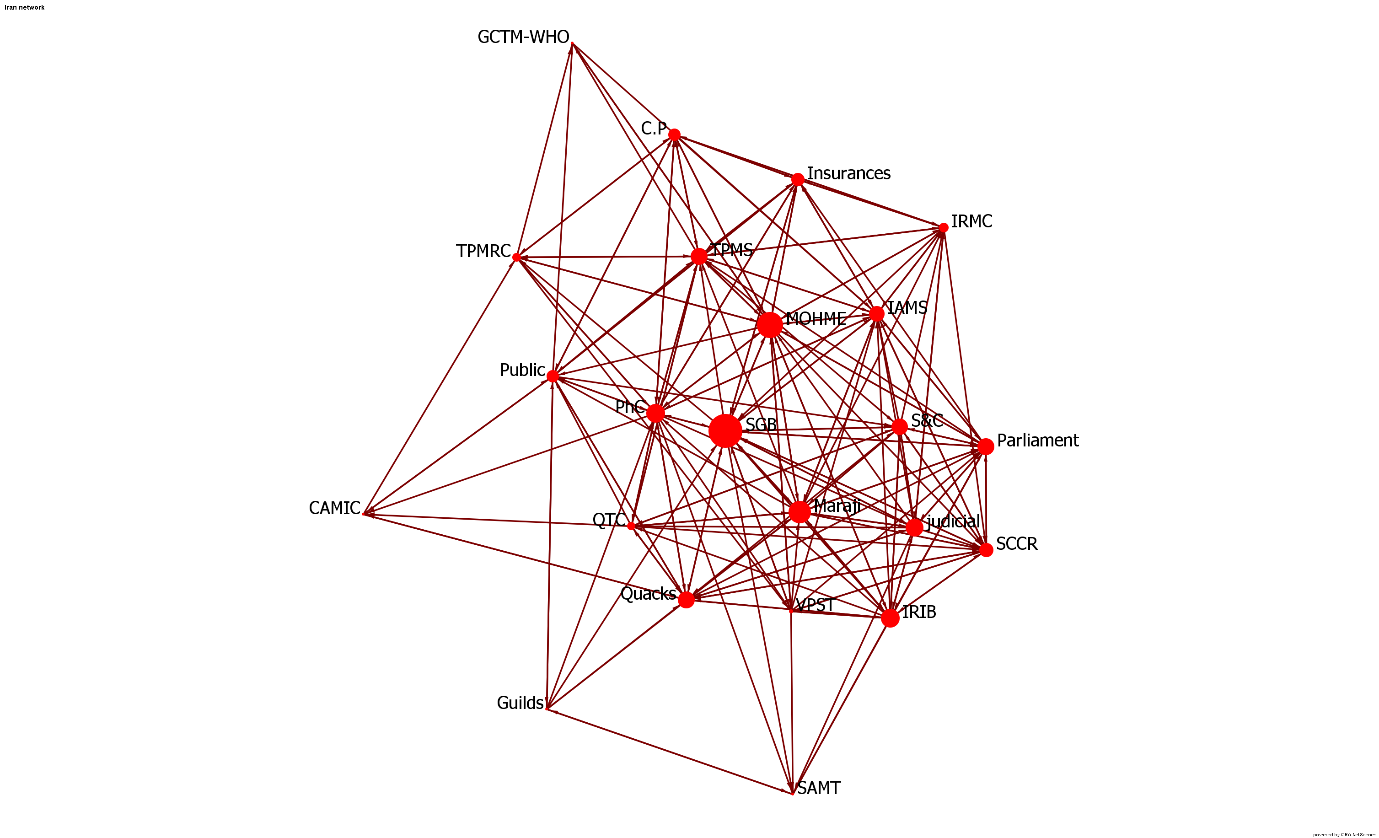
**
